# A *Drosophila* LexA Enhancer-Trap Resource for Developmental Biology and Neuroendocrine Research

**DOI:** 10.1534/g3.116.031229

**Published:** 2016-08-15

**Authors:** Lutz Kockel, Lutfi M. Huq, Anika Ayyar, Emma Herold, Elle MacAlpine, Madeline Logan, Christina Savvides, Grace E. S. Kim, Jiapei Chen, Theresa Clark, Trang Duong, Vahid Fazel-Rezai, Deanna Havey, Samuel Han, Ravi Jagadeesan, Eun Soo Jackie Kim, Diane Lee, Kaelina Lombardo, Ida Piyale, Hansen Shi, Lydia Stahr, Dana Tung, Uriel Tayvah, Flora Wang, Ja-Hon Wang, Sarah Xiao, Sydni M. Topper, Sangbin Park, Cheryl Rotondo, Anne E. Rankin, Townley W. Chisholm, Seung K. Kim

**Affiliations:** * Department of Developmental Biology, Stanford University School of Medicine, California 94305; †† Department of Medicine (Oncology Division), Stanford University School of Medicine, California 94305; † Phillips Exeter Academy, New Hampshire 03833; ** Science Department, Phillips Exeter Academy, New Hampshire 03833; ‡ Palo Alto High School, California 94306; § Pinewood School, Los Altos, California 94022

**Keywords:** *Drosophila melanogaster*, *Drosophila* neuro-endocrine system enhancer trap, LexA-LexAop binary expression system

## Abstract

Novel binary gene expression tools like the LexA-LexAop system could powerfully enhance studies of metabolism, development, and neurobiology in *Drosophila*. However, specific LexA drivers for neuroendocrine cells and many other developmentally relevant systems remain limited. In a unique high school biology course, we generated a LexA-based enhancer trap collection by transposon mobilization. The initial collection provides a source of novel LexA-based elements that permit targeted gene expression in the corpora cardiaca, cells central for metabolic homeostasis, and other neuroendocrine cell types. The collection further contains specific LexA drivers for stem cells and other enteric cells in the gut, and other developmentally relevant tissue types. We provide detailed analysis of nearly 100 new LexA lines, including molecular mapping of insertions, description of enhancer-driven reporter expression in larval tissues, and adult neuroendocrine cells, comparison with established enhancer trap collections and tissue specific RNAseq. Generation of this open-resource LexA collection facilitates neuroendocrine and developmental biology investigations, and shows how empowering secondary school science can achieve research and educational goals.

Differential gene expression in specific cells, at specific times and levels, is a principal driver of animal development and physiology. Research in *Drosophila melanogaster* has been invaluable for understanding the genetic basis of development and physiology. Based on strategies from bacterial genetics (Kroos and Kaiser 1984), investigators have developed transposon-based methods to detect enhancer activity (‘enhancer trapping’) following random insertion mutagenesis (O’Kane and Gehring 1987). The activity of *Drosophila* enhancer elements was first detected by the expression of randomly inserted *P*-elements carrying a weak promoter fused to a *lacZ* reporter gene (O’Kane and Gehring 1987).

To investigate and manipulate *Drosophila* gene expression in time and space, investigators have also exploited the activity of endogenous *cis*-regulatory enhancer elements to control expression of transactivators or repressors in specific temporal or spatial patterns of larval and adult tissues. Deployment of the yeast Gal4 transactivator to ‘drive’ expression of target genes fused to GAL4-responsive upstream activating sequences (UAS), established a binary gene expression system in *Drosophila* (Brand and Perrimon 1993; Hayashi *et al.* 2002; Gohl *et al.* 2011).

However, novel challenges in studying biological problems, like intercellular or interorgan communication, necessitate parallel manipulation of two, or more, independent cell populations (Rajan and Perrimon 2011). This requires additional binary expression systems independent of UAS-Gal4, such as the bacterial derived LexA system, which is based on LexA DNA binding domain:transactivator domain fusion proteins that regulate expression of transgenes fused to a LexA operator-promoter (LexAop; Szüts and Bienz 2000; Lai and Lee 2006; Pfeiffer *et al.* 2010; Knapp *et al.* 2015, Gnerer *et al.* 2015). The simultaneous use of two binary expression systems permits powerful epistasis experiments between different tissues (Shim *et al.* 2013), simultaneous clonal analysis of multiple cell populations (Lai and Lee 2006; Bosch *et al.* 2015), and visualization of specific physical cell–cell contacts (Gordon and Scott 2009; Bosch *et al.* 2015, Macpherson *et al.* 2015). However, successful use of combinations of binary expression systems depends largely on the availability of transgenic driver lines for specific developmental biology and physiology approaches. Within the framework of a high school science class developed in partnership between groups at Stanford University and Phillips Exeter Academy, we constructed the StanEx collection of LexA-based enhancer trap drivers for neuroendocrine and developmental biology research.

## Materials and Methods

### Generation of StanEx1 P-element

The StanEx enhancer trap *P*-element carries the weak P-promoter linked to a fusion of the LexA DNA binding domain-Gal4 hinge-Gal4 transcriptional transactivation domain (Pprom-LHG). To make pJFRC-MUH-70LHG70, a 3563 bp *Eag*I–*Eag*I fragment from pDPPattB-LHG (Yagi *et al.* 2010) was subcloned to the 7097 bp *Eag*I–*Eag*I fragment from pJFRC-MUH (Pfeiffer *et al.* 2010). To make pBS2KSP-attP-Pprom-GAL4-hsp70 3′UTR, a 3615 bp *Not*I–*Not*I fragment from pXN-attPGAL4LWL (Gohl *et al.* 2011) was subcloned to the *Not*I site on the pBS2KSP vector. To make pBS2KSP-attP-Pprom-LHG-hsp70 3′UTR, a 3563 bp *Eag*I–*Eag*I fragment from pJFRC-MUH-70LHG70 was Klenow filled-in, and ligated to a 3259 bp *Bam*HI–*Bam*HI fragment from pBS2KSP-attP-Pprom-GAL4-hsp70 3′UTR that was Klenow filled-in. A 3941 bp *Sac*II–*Xba*I fragment from pBS2KSP-attP-Pprom-LHG-hsp70 3′UTR (*Xba*I is methylated in the LHG coding region) was subcloned to the 8453 bp *Sac*II–*Xba*I fragment from pXN-attPGAL4LwL (Gohl *et al.* 2011). *P*-element vector transformation into a y[1],w[1118] strain was performed by standard procedures to generate the StanEx^1^ X-liked index insertion.

### Immunohistochemistry (IHC)

All tissues were fixed in 4% formaldehyde/PBS for 30 min, permeabilized in 0.2% Triton X-100/PBS for 4 hr, and blocked in 3% BSA/PBS for 1 hr. All antibody stainings were performed in 3% BSA/PBS, incubation of primary and secondary antibodies were overnight at 4°. PBS was used for all rinses and washes (3× each for primary and secondary antibody incubation steps). Antibodies used: Chicken anti-RFP 1:2000 (Rockland, 600-901-379). Goat anti-GFP 1:3000 (Rockland 600-101-215). Mouse anti-Tubulin 1:5000 (Sigma T5168). Donkey anti-Goat Alexa488 (Life Technologies, A11055). Donkey anti-Chicken Cy3 (Jackson ImmunoResearch 703-165-155). Donkey anti-Mouse Alexa594 (Life Technologies A21203). All secondary antibodies were used at 1:500. All samples were mounted in SlowFade Gold mounting medium with DAPI (Life Technologies, S36938).

### Microscopy

Microscopy was performed on a Zeiss AxioImager with filter sets 49, 38HE, 43HE, and 64HE for DAPI, Alexa488, Cy3, and Alexa 594, respectively, using the extended focus function. Confocal microscopy was performed using a Leica TCS SP5 using a Ti-Saphire multiphoton laser for DAPI, and the 488nm Argon, 546nm, and 594nm HeNe laser lines and HyD GaAsP detectors.

### Fly husbandry and fly strains

All fly strains were maintained on a standard cornmeal-molasses diet (http://flystocks.bio.indiana.edu/Fly_Work/media-recipes/molassesfood.htm). The following strains were used: y[1],w[1118] (Bloomington 6598), w[*]; ry[506] Sb[1] P{ry[+t7.2]= Δ2-3}99B/TM6B, Tb[1] (Bloomington 1798), w[*]; P{y[+t7.7] w[+mC]=26XLexAop2-mCD8::GFP}attP2 (Bloomington 32207), w[*]; L[*]/CyO; ftz[*] e[*]/TM6B,Tb[*],Antp[Hu]; *StanEx^1^* is the X-linked index insertion of the StanEx enhancer trap *P*-element collection utilizing the *P*-element promoter–LexA DNA binding domain “L”–Gal4 hinge region “H”–Gal4 transcriptional activation domain “G” construct (see above), y[1],w[1118], P{w[mC]=LHG]StanEx[1]}. We noted LexA-independent detection of 26x*LexAop2-CD8*::*GFP* in garland and pericardial nephrocytes at the L3 stage (Supplemental Material, Figure S6).

### Hybrid dysgenesis

Males of donor stock y,w,StanEx[1] were mated to w[*]; ry[506],Sb[1],Δ2-3/TM6B,Tb[1], and 10 F_1_ “jumpstarter” y,w,StanEx[1]; ry[506],Sb[1], Δ2-3/+ males were crossed to 20 double-balancer virgin females. *w+* F_2_ males were mated to w[*]; L[*]/CyO; ftz[*] e[*]/TM6B,Tb[*],Antp[Hu], the autosome of insertion was determined, and the insertion line stably balanced (Ryder *et al.* 2004). In the first iteration of the Bio470 class (see below), multiple *w*+ F_2_ males originating from the identical male jumpstarter parent were isolated into a stock leading to an artificially high rate of identical insertions. This practice was discontinued. One line with an X-chromosome insertion was isolated (*StanEx^AA10.1^*), despite our intercross scheme for exclusively isolating autosomally linked insertions (Table S1).

### Insertion site cloning

We followed an inverse PCR approach (Ochman *et al.* 1988, http://www.fruitfly.org/about/methods/inverse.pcr.html), to molecularly clone the insertion sites of StanEx *P*-elements. Overall, we sequenced genomic DNA adjacent to both 5′ and 3′ *P*-element sequences in 91% of the lines, and identified unique genomic sequence adjacent to at least one end of the *P*-element in the remaining lines. DNA restriction enzymes used: BfuCI (NEB R0636), *Hpa*II, (NEB R0171). Ligase used: T4 DNA Ligase (NEB M0202). Inverse PCR primer “Plac1” CAC CCA AGG CTC TGC TCC CAC AAT and “Plac4” ACT GTG CGT TAG GTC CTG TTC ATT GTT were used to clone genomic sequences off the 5′ end of the *P*-element. Inverse PCR primer “Kurt” TGT CCG TGG GGT TTG AAT TAA C and “Ulf” AAT ACT ATT CCT TTC ACT CGC ACT were used to clone genomic sequences off the 3′ end of *P*-element. Sequencing primer “Sp1” ACA CAA CCT TTC CTC TCA ACA A was used for 5′ end of the *P*-element. Sequencing primer “Berta” AAG TGG ATG TCT CTT GCC GA was used for the 3′ end of the *P*-element. For insertions where the sequence of one end only could be determined by inverse PCR, we pursued a gene-specific PCR approach (Ballinger and Benzer 1989) using *P*-element- and gene-specific primers. 5′-end-specific *P*-element primer “Chris”: GCA CAC AAC CTT TCC TCT CAA C. 3′-end-specific *P*-element primer “Dove”: CCA CGG ACA TGC TAA GGG TTA A. Line-specific primers sequences: LH4-5: CTTTGAGTACGCCCCACATTTG, RJ4-3: GCAAAATCTGATGACCCTGCTG, EM9-3: TGCCCAATCACTTGTGTCAAAA, DL5-3: TGTGTGAGTGTGCGAGTAAAGA, CS2-3ONE: ATGCAACACGTATTGGCACTTC, CS2-5: GAACAAGGTCAAGTGTCATCGC, CS2-3TWO: ATGAGCGCTTGAGATTCGGTAT, DRH4-3: TTGGGAAAGTCTACGGTGAGTG, IP1-3: GGAGCGAGATAAATACGAGGGG, IP3-3: AGTGGCGGGTTGAAACTAGAAT, SJH2-3: TGGGGAGTGTGAAATGTGCATA, AT5-3: TAGCTGACACCTGTTACCTTGG, AA14-5: AATTGCAATCGAATCGGGTTGG, AT1-5: CAGCTCGTTACGCAGGATTTTG, EH7-5: GCATTAGGTGGAGCTGCATTTC, EM7-3: GCCGAACGAGCAATTATACCAC, EM14-3: TTCTCTCCCAACCCAAACCAAA, EM15-3: GGAAAACTTCTCGCTGCAGTTT, EM16-3: AAGAAAGGAGGATGGCAAGGAG, JHW2-3: GACTCATTTGTTTCTGGTGGCC, JPC2-3: ACAATGCTGCAACACTTCTTCC, UT5-3: GTTGTAGTTGGTGGCGCATATC, TC1-5: AAGTATCCAAGCCAAGAAACCAC, TD1-3: CGGTTCGTTTACAATACGGCAG, and TD1-5: ACCTTATCAGAGCAGGAGAAAACC

A subset of inverse and direct PCRs was performed by LakePharma, Belmont, CA. Sequencing was performed by Sequetech Corp, Mountain View, CA.

### Clustering of tissue-specific patterns

Imaging data were digitized by tissue specific expression (0 = no expression, 1 = expression), and hierarchical clustering using Euclidian distance and complete linkage was performed using Cluster 3.0 software, and visualized deploying TreeView.

### Nomenclature of P-element insertion site

Independently of the direction of insertion, we defined the first nucleotide 3′ of the actual insertion into the genomic scaffold as the insertion site of the individual StanEx *P*-elements.

### Coursework at Phillips Exeter Academy and Stanford University

In the 11-wk spring term for 2013 and 2014, 12 students were selected for an elective advanced class called Bio470, with a prerequisite/corequisite of advanced placement (AP) biology or one term of a genetics elective. Bio470 was comprised of four scheduled 50-min periods, and a 70-min period, and ∼5–6 unscheduled hr per wk. This format transformed a standard biology classroom into an open laboratory. The course manual, weekly schedule, and problem sets are available on request. Problem sets, reading, and discussions covered transmission genetics using balancer chromosomes, the biology of mobile genetic elements, and methods including inverse PCR, molecular cloning, and antibody-based staining techniques. After learning basic *Drosophila* genetic methods, students spent ∼ 8–9 wk executing the hybrid dysgenesis crosses detailed in Figure S2. Mapping and balancer intercrosses ensued, in parallel with initial molecular mapping studies with PCR and DNA sequencing using standard genomic DNA recovery (see above). Intercrosses with *LexAop2-CD8*::*GFP* reporter strains were initiated in the last 3 wk, permitting instruction in larval dissection and microscopy to document tissue expression patterns of candidate enhancer traps. Refurbished Zeiss Axiophot microscopes were provided by S.K.K. and the department of Developmental Biology (Stanford) to Bio470. Based on performance in Bio470, two to three Exeter students, and high school students from Palo Alto, CA, or Los Altos, CA, were selected to continue studies in the Kim group at Stanford University School of Medicine during summer internships lasting about 6 wk. These studies included further molecular mapping of transposon insertion sites, and verification of tissue patterns of enhancer trap expression. Students returning in the fall term helped instructors to run the subsequent iteration of Bio470, and also pursued independent projects.

### Data and reagent availability

StanEx fly strains will be submitted to the Bloomington Stock Center. The course manual, weekly schedule, and problem sets are available on request. Figure S1 shows a schematic of the StanEx *P*-element. Figure S2 describes the hybrid dysgenesis crossing scheme. Figure S3 displays the clustering of tissue-specific expression data across StanEx lines. Figure S4 presents IHC analysis of StanEx enhancer traps in larval proventriculus. Table S1 contains the molecular and expression data of the StanEx enhancer trap collection. Table S2 summarizes StanEx enhancer trap lines analyzed for expression in adult flies.

## Results

### Generating a LexA-based enhancer trap collection

**Figure 1:**
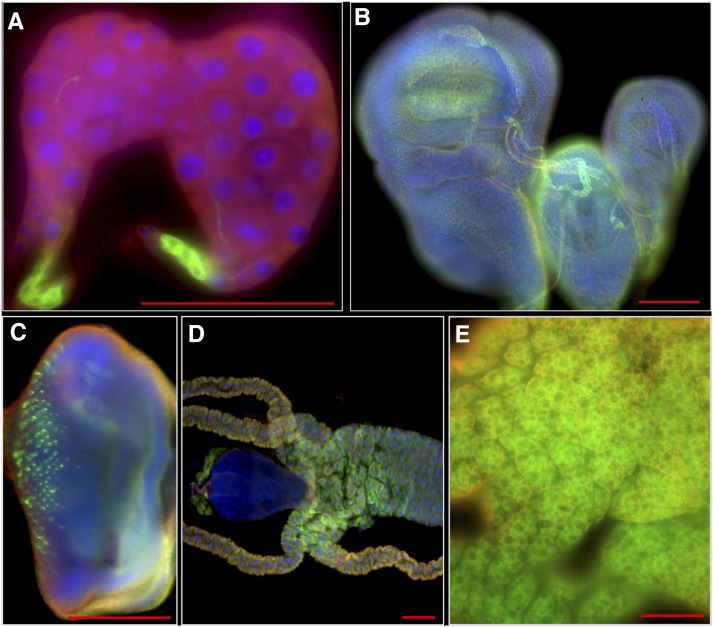
Expression pattern of *StanEx^1^* enhancer trap in tissues of wandering third instar larvae visualized by lexAop-CD8:GFP. This fly strain was used as a starter strain for the hybrid dysgenesis. For GFP channel only (green) see Figure S5. (A) CC cells in ring gland. (B) Expression in imaginal disc of wing, leg and haltere. (C) Eye disc. (D) Midgut. Note that expression in garland nephrocytes is lexAop-CD8:GFP background signal (see *Materials and Methods* and Figure S6). (E) Fat body. Green, Anti-GFP; Red, Anti-Tubulin; Blue, DAPI. Scale bar = 100 μm.

To build a LexA-based enhancer trap collection, we modified the InSITE *P*-element vector (Gohl *et al.* 2011) (Figure S1). A cDNA encoding the LexA DNA-binding domain fused to the hinge-transactivation domain of Gal4 (LexA::HG, Yagi *et al.* 2010) was inserted between the attP and loxP sites of the *P*-element vector (Figure S1, see *Materials and Methods*). This configuration sensitizes the *P*-element promoter expression to local genomic enhancer activity. This *P*-element vector also enables subsequent recombinase-mediated cassette exchange (RMCE; Gohl *et al.* 2011). We transformed an index X-chromosomal-linked fly strain, named *StanEx^1^* (Table S1: see *Materials and Methods*). Progeny from intercross of *StanEx^1^* with a line harboring a LexA operator- GFP reporter transgene (*LexAop2-CD8*::*GFP*; Pfeiffer *et al.* 2010) had clear membrane**-**associated GFP expression in several tissues including ring gland, imaginal discs of the wing, eye, haltere and T3 leg, eye imaginal disc, midgut, and fat body (Figure 1 and Figure S5), confirming the suitability of *StanEx^l^* as a starter line for transposase-mediated hybrid digenesis. We then mobilized the *StanEx^1^* *P*-element insertion to autosomes using standard hybrid dysgenesis methods, to generate LexA *P*-element insertion lines (Figure S2) (see *Materials and Methods*) (O’Kane and Gehring 1987; Pfeiffer *et al.* 2008). Using *StanEx^1^* *P*-element mobilization we obtained 149 initial lines.

### Mapping LexA P-element insertion sites

**Figure 2:**
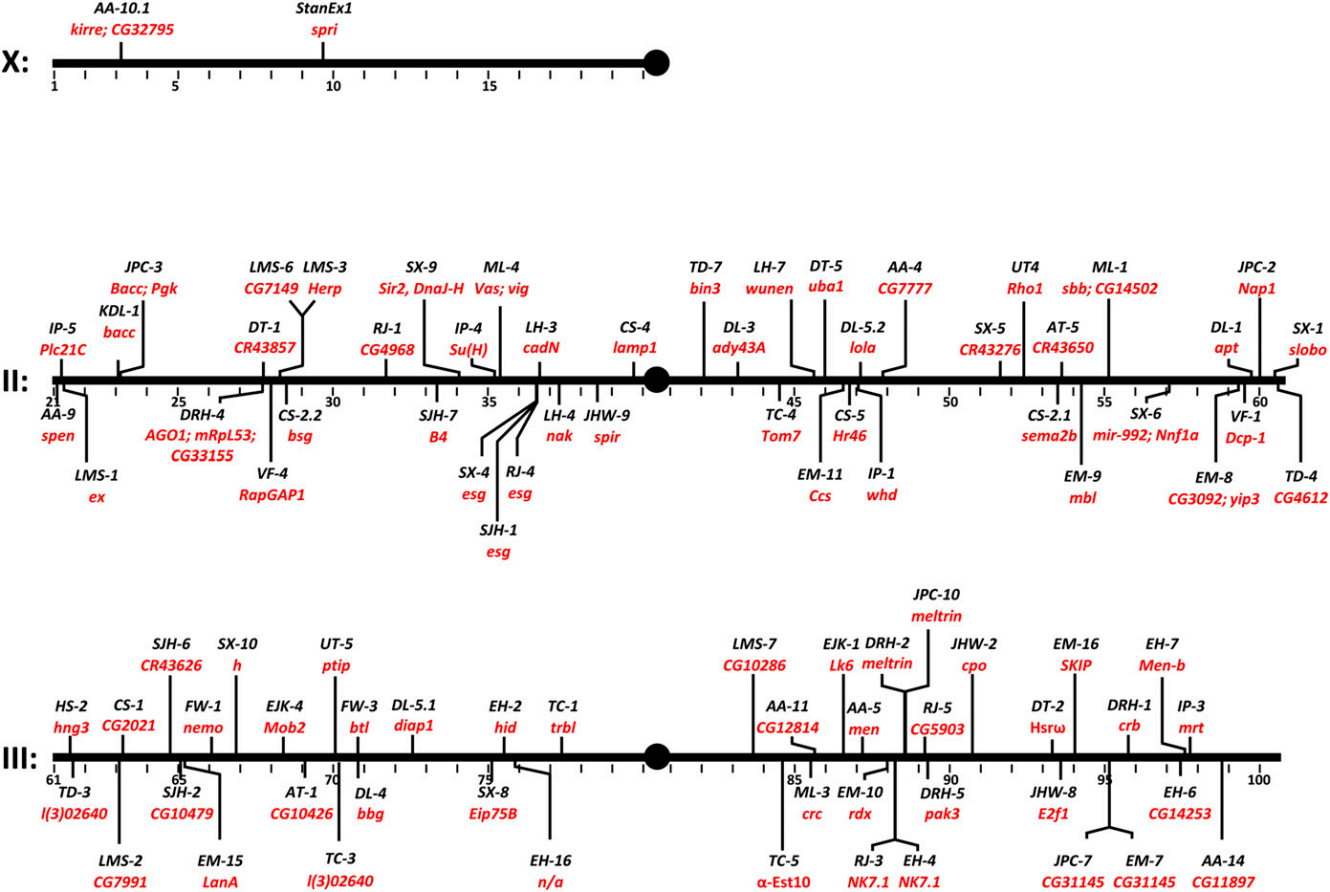
Distribution of novel StanEx LexA::HG enhancer trap insertions sites in chromosomes I, II and III. See Table S1 for corresponding detailed data. Multiple insertions have been obtained in esg, NK7.1, CG31145, Meltrin, and bacc.

Standard molecular methods were used to map the chromosomal insertion position (Figure 2 and Table S1) (http://stanex.stanford.edu/search/index.php). After eliminating lines with identical insertions (see *Materials and Methods*), we identified 93 lines with a unique insertion position (Table S1). The insertions were distributed across the autosomes, with each arm of chromosomes 2 and 3 receiving around a quarter of the insertions (2L, 23 insertions; 2R, 23 insertions; 3L, 20 insertions; 3R, 24 insertions). Three insertions were linked to repetitive sequences, precluding mapping of the chromosomal integration site (*StanEx^DT3^*, *StanEx^AA2^*, and *StanEx^FW4^*).

The majority of insertions were linked to specific genes, including many developmental regulators. Three independent insertions (*StanEx^SX4^*, *StanEx^SJH1^*, and *StanEx^RJ4^*) (Table S1) were located in the region 5′ of the transcriptional start site of *escargot* (esg), a known ‘hot-spot’ for *P*-element insertion (Bellen *et al.* 2004; Hayashi *et al.* 2002). Two insertions mapped within 4.5 kb at the locus encoding *Meltrin* (*StanEx^JPC10^* and *StanEx^DRH2^*), and two insertions mapped within the first intron of *CG31145* (*StanEx^JPC7^* and *StanEx^EM7^*). We also recovered two insertions in *NK7.1*, which showed reverse orientation (*StanEx^RJ3^* and *StanEx^EH4^*). Overall, mapping of the 93 StanEx *P*-element insertions revealed a strong bias for insertion in the 5′ end of genes: 72% of all insertions were mapped to within 300 bp of the 5′ regulatory sequence preceding the transcriptional unit (41%) or its first exon (32%). In one line, the *P*-element inserted in the region distal to the 3′ end of the nearest gene (*StanEx^SX-5^*, inserted near *CR43276*). 69% of the StanEx insertions mapped to loci previously shown to harbor five or more *P*-elements insertions within +/– 100 bp (Bellen *et al.* 2011). To our knowledge, LexA lines or other LexA-based tools have not been described previously for these 64 loci. Further, we isolated an additional 17 lines in which *StanEx^1^* inserted in unique sites with no known *P*-element insertions within this radius. Some of these unique insertions include developmentally important genes, such as *NK7.1*, *ptip*, *Tom7*, *mir-992/Nnf1a*, *CG7149*, *CadN*, *CG31145*, *Meltrin*, *nemo*, *CG3092/yip3*, *rdx*, *W*, and *bsg (intergenic)*. Thus, our approach generated multiple novel LexA-based autosomal enhancer traps.

### Tissue expression of LexA in the StanEx collection

**Figure 3:**
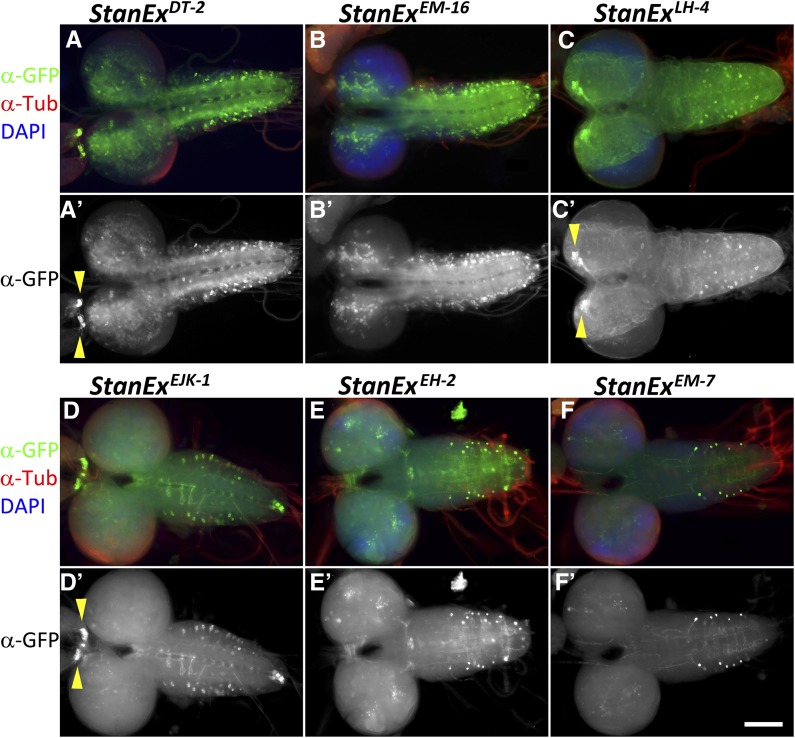
Immunohistochemical analysis of individual StanEx enhancer trap line expression in larval brain and VNC. Third larval instar CNS and VNC expression of LexA::HG is visualized by *LexAop-CD8*::*GFP*. (A, A′) *w*; *StanEx^DT-2^/ LexAop-CD8*::*GFP*. Arrowheads in A′ mark CC cells. (B, B′) w; StanEx^EM-16^/ LexAop-CD8::GFP. (C, C′) *w*; *StanEx^LH4^/+*; *LexAop-CD8*::*GFP/+*. Arrowheads in C′ mark IPCs. (D, D′) *w*; *StanEx^EJK-1^/LexAop-CD8*::*GFP*. Arrowheads in D′ mark CC cells. (E, E′) w; StanEx^EH-2^/LexAop-CD8::GFP. (F, F′) w; StanEx^EM-7^/LexAop-CD8::GFP. Green, Anti-GFP; Red, Anti-Tubulin; Blue, DAPI. Scale bar = 100 μm.

**Figure 4:**
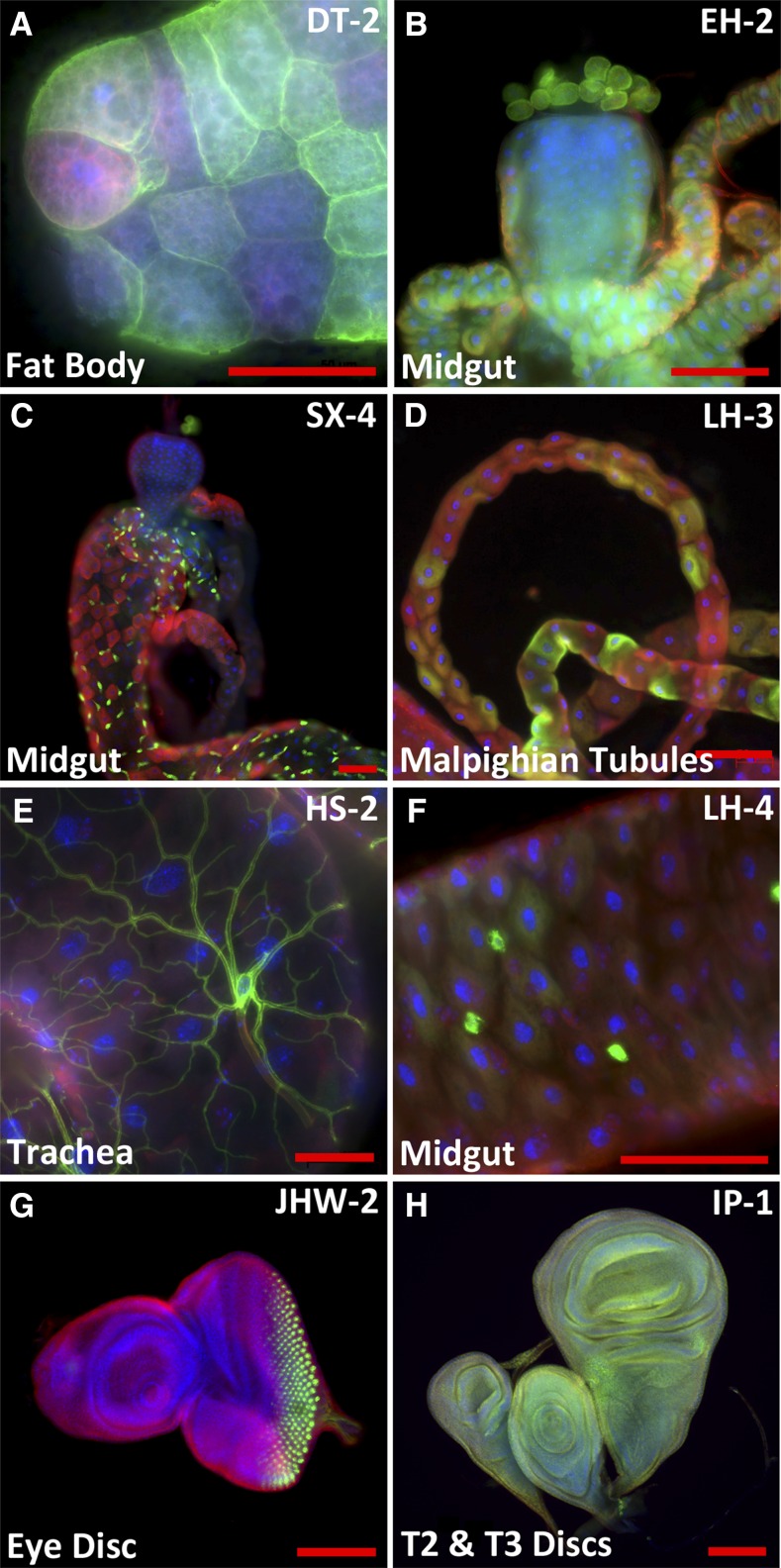
Immunohistochemical analysis of StanEx enhancer trap expression in third instar larval tissue visualized by *LexAop-CD8*::*GFP*. (A) *w*; *StanEx^DT-2^/LexAop-CD8*::*GFP*. Expression in fat body. Note the variable expression in fat body cells. (B) *w*; *StanEx^EH-2^/LexAop-CD8*::*GFP*. Expression in anterior midgut. Note that expression in garland nephrocytes is lexAop-CD8:GFP background signal (see *Materials and Methods* and Figure S6). (C) w; StanEx^SX-4^/+; LexAop-CD8::GFP/+. Expression in midgut. (D) *w*; *StanEx^LH-3^/+*; *LexAop-CD8*::*GFP/+*. Expression in malphigian tubules. Note the variable expression in individual cells. (E) *w*; *StanEx^HS-2^/LexAop-CD8*::*GFP*. Expression in trachea located on midgut. (F) *w*; *StanEx^LH-4^/LexAop-CD8*::*GFP*. Expression in small cells in midgut consistent with expression patterns of entero-endocrine cells. (G) *w*; *StanEx^JHW-2^/LexAop-CD8*::*GFP*. Expression in photoreceptor clusters in third instar eye disc. (H) *w*; *StanEx^IP-1^/+*; *LexAop-CD8*::*GFP/+*. Expression in third instar haltere, leg, and wing disc. Green, Anti-GFP; Red, Anti-Tubulin; Blue, DAPI. Scale bar = 50 μm.

To evaluate the tissue expression patterns of the insertion lines, we intercrossed the *LexA*::*HG* transcriptional activator insertion lines to flies harboring a *LexAop2-CD8*::*GFP* reporter (Pfeiffer *et al.* 2010). Third instar larvae of bitransgenic offspring were analyzed by IHC staining for GFP expression using a counterstain for microtubules (anti-tubulin) and cell nuclei (DAPI). Image data from 91 LexA lines were collected and organized into a searchable public database (see below). Within the collection, we detected expression in nearly all tissues of the L3 larva, including a variety of neuronal cell types in the central nervous system (CNS), ventral nerve cord (VNC) (Figure 3) and peripheral nervous system (PNS), imaginal discs, and a wide range of other somatic tissues like fat body, malpighian tubules, and trachea (Figure 4). We also observe LexA expression in a subset of cells in the midgut with features of gut stem cells (Figure 4C), *StanEx^SX4^*, inserted in escargot (Korzelius *et al.* 2014), and entero-endocrine cells (*StanEx^LH4^*) (Figure 4F) inserted in *numb-associated kinase* (Takashima *et al.* 2011).

**Figure 5:**
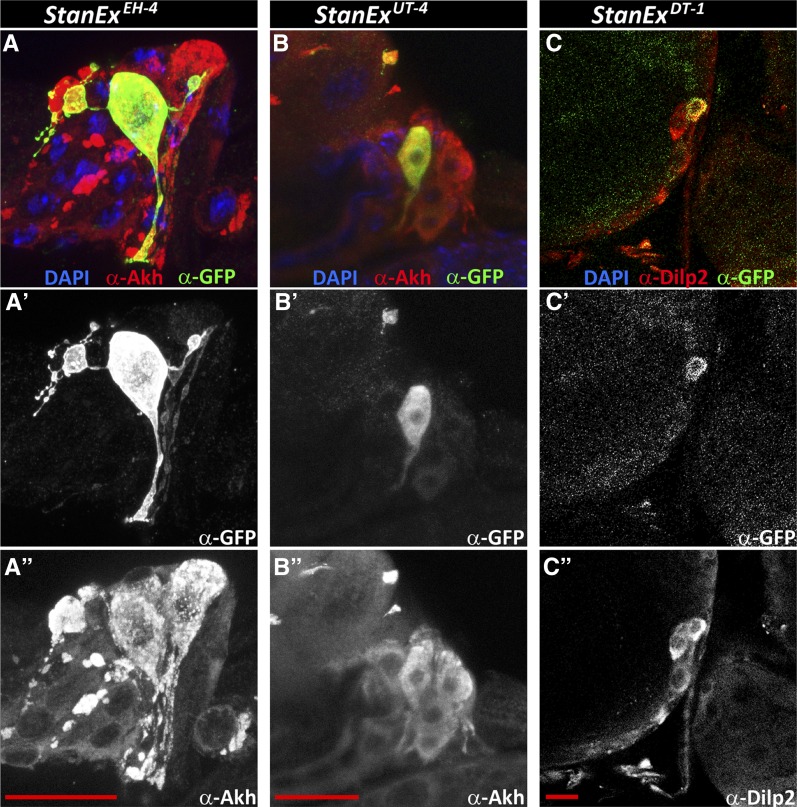
Heterogeneous enhancer trap expression in endocrine CC cells and IPCs. (A–A′′) *w*; *StanEx^EH-4^ /LexAop-CD8*::*GFP*. Green, Anti-GFP; Red, Anti-Akh, marking all CC cells; Blue, DAPI. (A′) Anti-GFP-channel only. (A′′) Anti-Akh channel only. (B–B′′) *w*; *StanEx^UT-4^/+*; *LexAop-CD8*::*GFP/+*. Green, Anti-GFP; Red, Anti-Akh, marking all CC cells; Blue, DAPI. (B′) Anti-GFP-channel only. (B′′) Anti-Akh channel only. (C–C′′) *w*; *StanEx^DT-1^/+*; *LexAop-CD8*::*GFP/+*. Green: Anti-GFP, Red: Anti-Dilp2, marking all IPC cells, Blue: DAPI. (A′) Anti-GFP-channel only. C′′) Anti-Dilp2 channel only. Scale bar in A′′, B′′ and C′′ = 20 μm.

To facilitate further comparison of the StanEx collection lines to other expression data sets, we analyzed a subset of 76 StanEx lines that are unambiguously inserted within, or adjacent to, a single known gene. On average, each StanEx line expressed LexA activity in five distinct cell types (Figure S3). One line expressed in a single tissue only (*StanEx^FW3^*). These findings are consistent with prior studies indicating that enhancers only very rarely produce expression patterns limited to a single cell type in a complex organism (Jenett *et al.* 2012). In three lines we did not detect any discernible GFP expression, indicating the absence of inherent LexA expression from these StanEx *P*-element insertions (StanEx^DL-3^, StanEx^DRH1^, and StanEx^VF1^). We reproducibly detected LexA expression in neuronal cells of the CNS in 84% of lines (64/76) and in the VNC of 83% (63/76) (Table S1). This includes median protocerebral insulin-producing cells (IPCs) (Figure 3C and arrowheads in Figure 3C′), a group of cells neighboring IPCs, CNS commissural neurons, CNS neurons in the optic lobes, and CC (Corpora Cardiaca) cells (arrowheads in Figure 3, A′ and D′). Many lines expressed unique, cell-specific expression patterns. For example, in four StanEx insertion lines we observed reporter expression in a subset of CC cells (Figure 5), a pattern of mosaic expression not previously described to our knowledge (Park *et al.* 2011). Seven out of 95 StanEx lines drove reporter gene expression in the proventriculus, a larval foregut structure (Figure S4). We observed LexA-dependent labeling of distinct proventricular cell subsets in each of these StanEx lines, including subsets of anterior, medial, and posterior ‘stripes’ in the outer visceral mesoderm, the inner epithelial layer, and the cardiac valve. Patterned gene expression in the proventriculus has been described (Singh *et al.* 2011; Josten *et al.* 2004; Senger *et al.* 2004), and the novel binary expression resource created here could be useful for studying mechanisms underlying patterning of the proventriculus.

To assess commonalities of expression patterns in the StanEx collection, we performed clustering analysis of the digitized tissue expression pattern of all StanEx lines (Figure S3) (see *Materials and Methods*). This revealed covariation between the identified tissue expression domains. For example, with one exception (*StanEx^KDL-1^*, insertion in *bacchus*), all lines expressing in the VNC showed expression in the CNS. Conversely, all CNS expressing lines except two (*StanEx^DRH-2^*, insertion in *meltrin*, *StanEx^SX-10^*, insertion in *hairy*) had detectable expression in the VNC. These findings support the prior suggestion of a ‘linked’ enhancer code shared by these two tissues (Li *et al.* 2014).

To facilitate storage of imaging and molecular data, including image archiving, annotation, retrieval, and database mining, we generated the StanEx website (http://stanex.stanford.edu/search/index.php; L. Huq, L. Kockel, and S. K. Kim, unpublished data), an online database searchable by expression pattern, cytology, and specific genes. This includes supplementary image analysis, data from immunostaining, and molecular features of StanEx insertion loci, and is freely accessible for the scientific community. Although database mining is beyond the scope of this report, we present examples below that illustrate the types of studies our data permit.

### Comparison of StanEx enhancer trap tissue expression patterns with prior data sets

To verify the quality of our image and histological analysis, we compared analysis from prior data sets reporting tissue-specific RNA expression to reporter-gene expression patterns generated with the StanEx LexA enhancer trap collection. Specifically, we used stage-specific and organ-specific RNA-seq data recently obtained from brain, imaginal discs, digestive system, fat body, and salivary gland (Graveley *et al.* 2011) to query a subset of 71 StanEx lines inserted within a specific gene. Overall, 98% StanEx lines partially or fully reproduced tissue-specific expression detected previously by RNA-Seq. We also compared the tissue expression pattern of our collection with that from a previously described Gal4 enhancer trap collection (NP) of the *Drosophila* Genome Resource Center (DGRC; Hayashi *et al.* 2002). The reported expression pattern of 73% of these NP enhancer traps fully or partially matched the gene expression pattern reported in the RNAseq dataset (Graveley *et al.* 2011). We found that 52 genes with 274 Gal4-based insertions in the NP collection are also represented in the LexA-based StanEx collection. Direct comparison of the reported expression patterns of NP and StanEx insertions revealed a 91% full or partial overlap. Thus, our analyses indicate good concordance between StanEx enhancer trap expression, and tissue patterns of gene expression derived from RNA-Seq or enhancer trap collections data sets.

### Neuroendocrine cell enhancer traps in the StanEx collection

To identify additional uses of the StanEx collection, we focused on drivers for neuroendocrine cells. For example, the IPCs are neuroendocrine cells that produce and secrete the hormone insulin to regulate carbohydrate homeostasis and growth. Complementary to this, CC cells secrete the polypeptide hormones Akh and Lst, to mobilize energy reserves and regulate insulin secretion (Kim and Rulifson 2004; Alfa *et al.* 2015). Experimental dissection of the neuroendocrine cellular circuitry orchestrating hormonal regulation of metabolism should greatly benefit from independent binary LexA-LexAop and GAL4-UAS genetic systems. We identified 47 StanEx enhancer traps that drove reporter gene expression in the ring gland of third instar larvae. Of these 47 lines, 37 drove reporter-gene expression in CC cells. In addition, IPCs in the pars intercerebralis of the *Drosophila* brain are marked by 13 lines (Table S1). A previous study (Harvie *et al.* 1998) that analyzed the ring gland expression of 510 PZ enhancer traps found 76 lines (15%) showing ring gland expression. An analysis of a subset of these 76 lines revealed three lines with CC cell expression. However, the small sample size of molecularly characterized PZ lines (12/76, 16%) precluded comparison between tagged genes in the two collections.

CC cells undergo extensive remodeling during metamorphosis but persist in adults, with connections to the foregut and heart (Alfa *et al.* 2015; Cognini *et al.* 2011), while other cells in the larval ring gland comprising the prothoracic gland or corpus allatum degenerate. To determine if neuroendocrine LexA expression persists after metamorphosis, we analyzed LexA enhancer trap expression produced by adult CC cells in a subset of StanEx lines. We used a dual labeling strategy, marking adult CC cells with akh-G4, UAS-CD4:tandemTomato (Park *et al.* 2011; Han *et al.* 2011), and tested if LexA::HG directed expression of the reporter *LexAop2-CD8*::*GFP* (Pfeiffer *et al.* 2010). In seven of 13 (54%) StanEx lines that expressed LexA in larval CC cells, we observed maintenance of LexA expression in adult CC cells. By comparison, 28% of the so-called FlyLight enhancer constructs expressed in larval neurons continued to be expressed in adult neurons (Li *et al.* 2014). Using a similar strategy for IPCs, we found that one of seven StanEx lines (*StanEx^DL-5.1^*, insertion in Diap1) maintained LexA::HG expression in adult IPC cells, consistent with prior studies of larval and adult IPCs (Jenett *et al.* 2012). In three StanEx lines, we reproducibly observed labeling of IPC and CC cell subsets (*StanEx^EH-4^*, insertion in *NK7.1*, *StanEx^UT-4^*, insertion in *Rho1*, *StanEx^DT-1^*, insertion close to *CR43857*, Figure 5). Thus, our findings provide evidence for heterogeneous gene expression in individual IPCs and CC cells, supporting the view that these cells may have diversified function (Kim and Neufeld 2015; Rajan and Perrimon 2012). The ability to discriminate individual cells within a cluster should prove useful for studies of dynamic synapse development or remodeling, a possibility previously raised in other neurotransmitter or hormone-producing cell types (De Paola *et al.* 2006).

## Discussion

Here, we used transposase-mediated *P*-element mobilization to trap enhancers that express a chimeric LexA::HG fusion. We generated a collection of *Drosophila* lines that should prove useful for genetic, developmental, and physiological studies of cells and tissues. The ability to use LexA::HG in combination with other binary systems, like UAS-GAL4, should advance studies of short-range and long-range cell interactions and interorgan signaling *in vivo*, a growing area of investigation in *Drosophila*. The resources described here should prove valuable for a range of investigations, in particular for neuroendocrine research, and were generated from two consecutive iterations of a high school biology course. This illustrates the feasibility of building partnerships between research universities and secondary schools to conduct biological research with practical outcomes.

*P*-element insertion in flies is nonrandom (O’Hare and Rubin 1983; Berg and Spradling 1991), with a strong bias for transposition to the 5′ end of genes (Spradling *et al.* 1995). *P*-element insertion preferences are likely guided by the chromatin state, and other structural features of the target DNA, rather than a sequence-based DNA motif (Liao *et al.* 2000). We find a similar preference in the StanEx *P*-element, with ∼72% of the insertions in the promoter or 5′ UTR regions of genes. Similar to outcomes from studies of KG element mobilization (Bellen *et al.* 2004), we note that the sites of multiple *P* element insertions (hotspots) in our study were within one cytological unit of breakpoints in the CyO and TM6B chromosomes, balancer chromosomes used in our hybrid dysgenesis protocol, indicating points of greater chromosome accessibility.

The LexA::HG StanEx enhancer traps display a significant degree of overlapping expression patterns when compared to Gal4 enhancer traps inserted near the same site, and also overlap significantly with existing RNAseq data (Hayashi *et al.* 2002; Graveley *et al.* 2011). Hence, the weak *P*-element promoter linked to the LexA::HG reporter of the StanEx1 *P*-element represents a reliable enhancer trap. Multiple StanEx lines revealed distinct expression patterns in many developmentally and physiologically key cell populations and tissues. For example, we isolated several LexA enhancer traps driving expression in neuroendocrine cells like IPCs and CC cells, including enhancer traps reproducibly expressed in subsets of IPCs or CC cells. Based on the expression of secreted neuropeptides, previous reports have indicated a subdivision within the IPC neuroendocrine cell clusters (Kim and Neufeld 2015). However, genetic elements permitting targeting of IPC or CC cell subsets have not been previously available, to our knowledge. The new genetic tools described here should enable the further analysis of the IPC–CC inter-relationships, and foster characterization of possible cell diversification within these neuroendocrine clusters. Several StanEx lines also show unique expression patterns in the proventriculus of the third instar larva. The proventriculus is an organ derived from at least three tissue layers, visceral mesoderm, ectodermal epithelial layer and the cardiac valve (Pankratz and Hoch 1995). We observed reporter expression restricted in antero-posterior stripes, in both inner and outer cell layers of the proventriculus. Restricted expression patterns in the proventriculus have been noted for genes encoding GATA factors (Senger *et al.* 2006), the dve transcription factors (Kölzer *et al.* 2003) and STAT92E (Singh *et al.* 2011). To our knowledge, few layer- and pattern-specific genetic tools have been reported for this organ, and none based on LexA.

We observed a high degree of partial or full overlap between the enhancer trap activity displayed by the individual StanEx insertion lines and the respective counterparts of the NP Gal4 enhancer traps (Hayashi *et al.* 2002), and the reported mRNA expression pattern of the gene into which the StanEx lines are inserted (Graveley *et al.* 2011). We suspect that differences in reported expression patterns might be due to inherent technical limitations of a bigenic expression system, including delayed production of the GFP reporter protein, or variable sensitivity of the minimal *P*-element promoter in the StanEx enhancer trap element to endogenous enhancers. The regional differences in *P*-element insertion between the NP Gal4 lines and StanEx enhancer traps within the same gene might represent another factor to account for the observed differences of expression.

The LexA-LexAop binary expression system in the StanEx enhancer trap collection provides opportunities for a variety of intersectional methods with the UAS-Gal4 expression system (Shim *et al.* 2013; Lai and Lee 2006; Gordon and Scott 2009; Bosch *et al.* 2015). One advantage for combining these methods is the lack of interfering cross-talk between the LexA-LexAop and Gal4-UAS system. The transcriptional cross-activation of Gal4 to LexAop promoters, and of the LexA transcriptional activator on UAS regulatory sequences is minimal, consistent with the independent binding-site specificity of the two systems (Lei and Lee 2006). With the advent of φC31-attP mediated transformation (Groth *et al.* 2004), large collections of promoter fragment-driven Gal4 transgenes have been generated at specific attP sites (Jenett *et al.* 2012). However, the pairing of somatic chromosomes has been shown to give rise to cross-regulation (transvection) of enhancer/promoter elements between homologous chromosomes (Kassis 2012; Mellert and Truman 2012; Bateman *et al.* 2012). Combination of sister chromosomes harboring distinct transgenes transformed into the same attP site, *e.g*., promoter1-Gal4 and promoter2-LexA, might trigger transvection, which can severely confound experimental outcomes. By contrast, the random integration of a LexA-containing StanEx enhancer trap will likely be less prone to transvection when used in combination with Gal4, as long as the integration sites of the two transgenes differ significantly. The StanEx enhancer trap collection should complement ongoing projects to generate LexA driver lines with enhancer fusions with site-specific insertion (Jenett *et al.* 2012), and constitutes a valuable experimental tool resource. The creation of a searchable online StanEx database will enable the scientific community to select the strain of choice, and the associated fly strains (including stocks generated in future iterations of the course) will be available in the public fly stock repositories.

The results, resources, and experience detailed here stem from consecutive iterations (2013–2014) of a high school biology course now in its 5th yr of enrollment. Fruit fly genetics and developmental biology served as an ideal vehicle for building an authentic, open-ended research program for new scientists, based on key attributes (see *Materials and Methods*) including: (1) relative technical simplicity of fly husbandry; (2) conceptual simplicity requiring only modest prior mastery of biology and genetics, transitioning to complex operations like tissue dissection, histology, microscopy, and code-writing to create the StanEx database; (3) compatibility with flexible scheduling; (4) concrete achievement milestones for both instructors and students; (5) project ownership; (6) publishable results; and (7) cost feasibility in the setting of a modern genetics curriculum (Redfield 2012). Our experimental strategies had the advantage of offering each student a reasonable prospect of isolating one or more novel fly strains, thereby promoting a sense of discovery and ownership (Hatfull *et al.* 2006), a key research and educational goal. We recognize that the timeframe of the course limited opportunities for experimental design by students. However, advantages from this compression included a requirement for a parallel project structure transitioning to longitudinal studies (one class continues work from the preceding class). This continuity enhanced opportunities for students to mentor peers, and to interact with instructors as colleagues. For adult instructors, the course offered unusual opportunities for career development outside a more traditional classroom setting. Fruit fly genetics has been previously used to introduce research to a large consortium of undergraduates (Call *et al.* 2007). Our experience demonstrates that longitudinal studies involving multi-generational genetics, animal husbandry, molecular biology, immunohistochemistry and bioinformatics can thrive in a secondary school setting.

## 

## Supplementary Material

Supplemental Material
