# Supplementary material for: A Drosophila LexA Enhancer-Trap Resource for Developmental Biology and Neuroendocrine Research
Source: G3 (Bethesda). 2016 Aug 15;6(10):3017–26. doi: 10.1534/g3.116.031229 (PMC5068927; doi:10.1534/g3.116.031229)
Supplement: Supplemental Material [file supp_6_10_3017__index.html]

A Drosophila LexA Enhancer-Trap Resource for Developmental Biology and Neuroendocrine Research — Supplemental Material 

# A *Drosophila* LexA Enhancer-Trap Resource for Developmental Biology and Neuroendocrine Research

## Supplemental Material for Kockel, *et al*, 2016

**Files in this Data Supplement:**

- Figure S1 - Schematic map of molecular modules composing the StanEx P-element. (.tif, 885 KB)
- Figure S2 - Hybrid dysgenesis crossing scheme. (.tif, 1177 KB)
- Figure S3 - Clustering of tissue specific expression data across StanEx lines. (.tif, 1109 KB)
- Figure S4 - Immuno-histochemical analysis of patterned StanEx enhancer trap expression in third instar larval proventriculus visualized by *lexAop-CD8::GFP*. (.tif, 4731 KB)
- Figure S5 - Expression pattern of StanEx1 enhancer trap in tissues of wandering third instar larvae visualized by lexAop-CD8:GFP. (.tif, 761 KB)
- Figure S6 - Detection of GFP signal in *lexAop2-CD8::GFP* independently of LexA driver. (.tif, 337 KB)
- Table S1 - Summary table of molecular data and expression characterization of the StanEx enhancer trap collection. (.xlsx, 243 KB)
- Table S2 - Summary of StanEx enhancer trap lines analyzed for adult IPC and CC cell expression. (.xlsx, 26 KB)
