# Supplementary figures and images for: A Drosophila LexA Enhancer-Trap Resource for Developmental Biology and Neuroendocrine Research
Source: G3 (Bethesda). 2016 Aug 15;6(10):3017–26. doi: 10.1534/g3.116.031229 (PMC5068927; doi:10.1534/g3.116.031229)

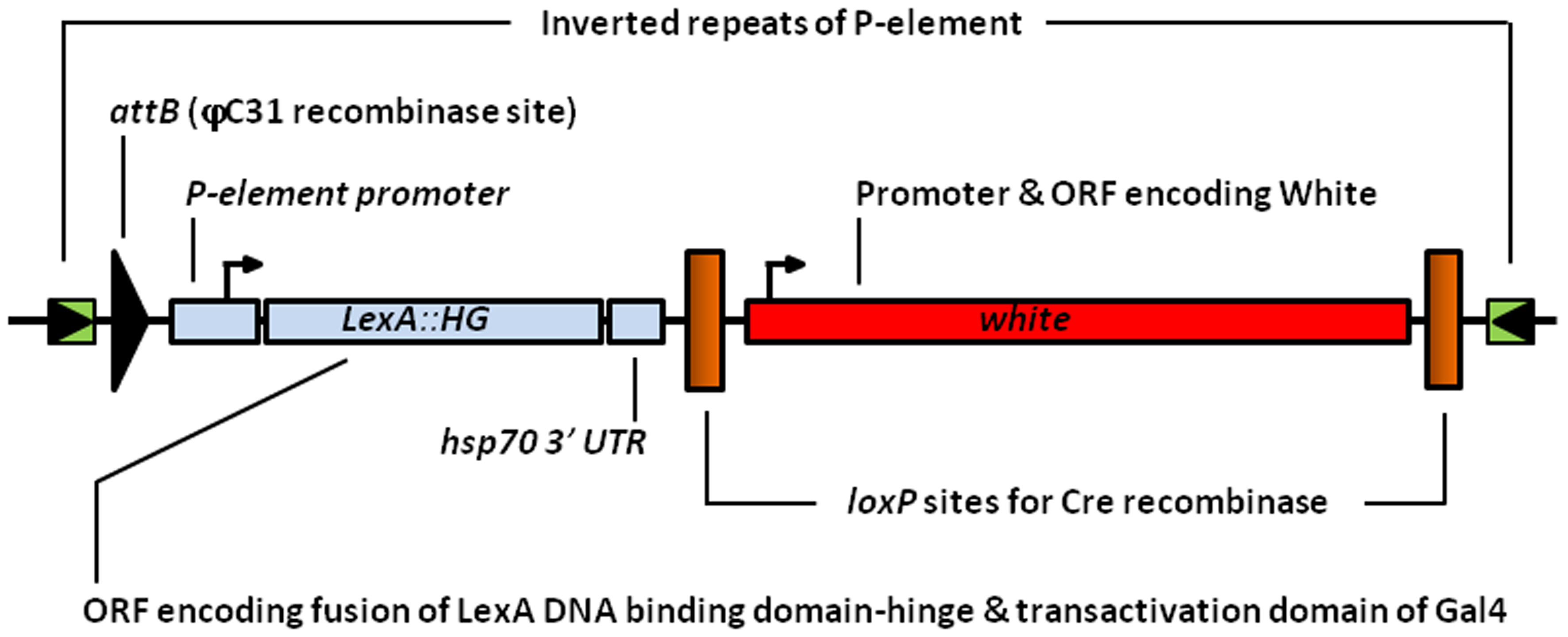

Supplement: Supplemental Material [file supp_g3.116.031229_FigureS1.tif]

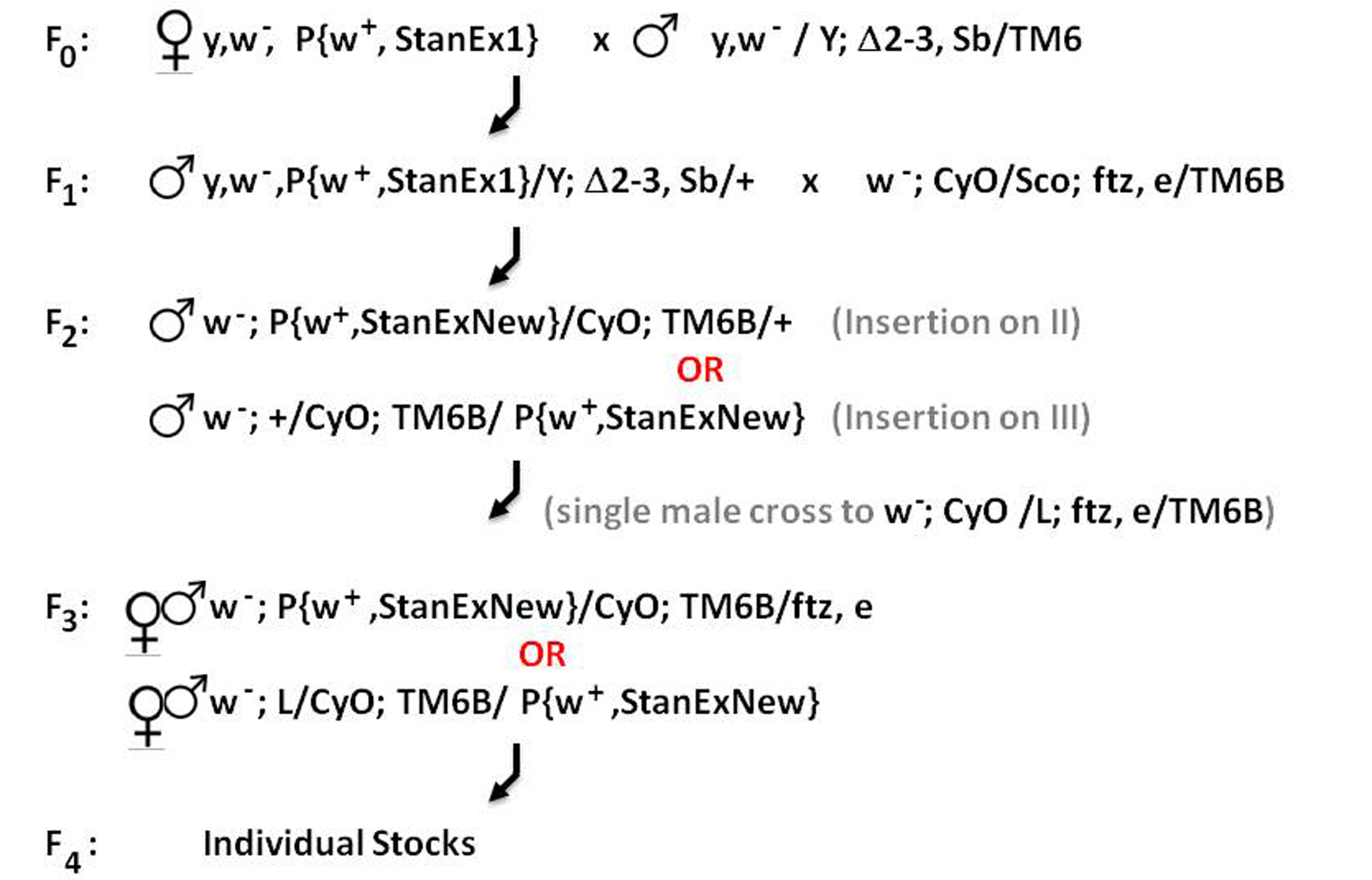

Supplement: Supplemental Material [file supp_g3.116.031229_FigureS2.tif]

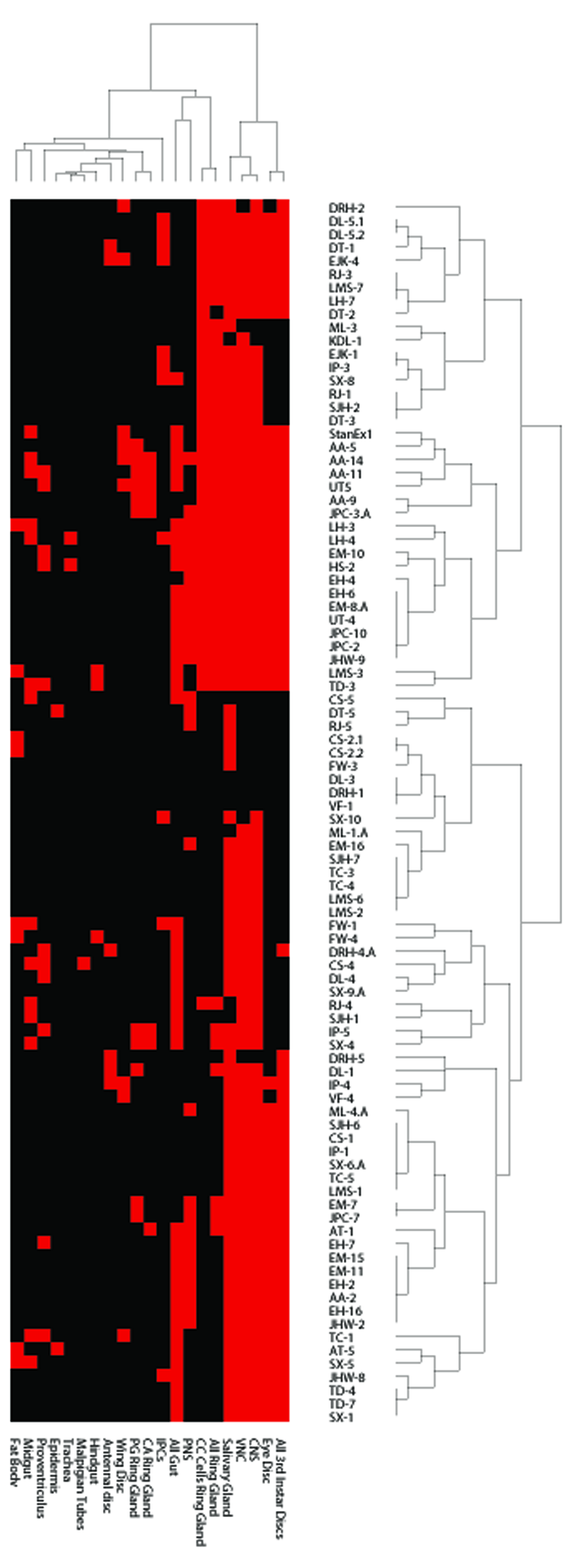

Supplement: Supplemental Material [file supp_g3.116.031229_FigureS3.tif]

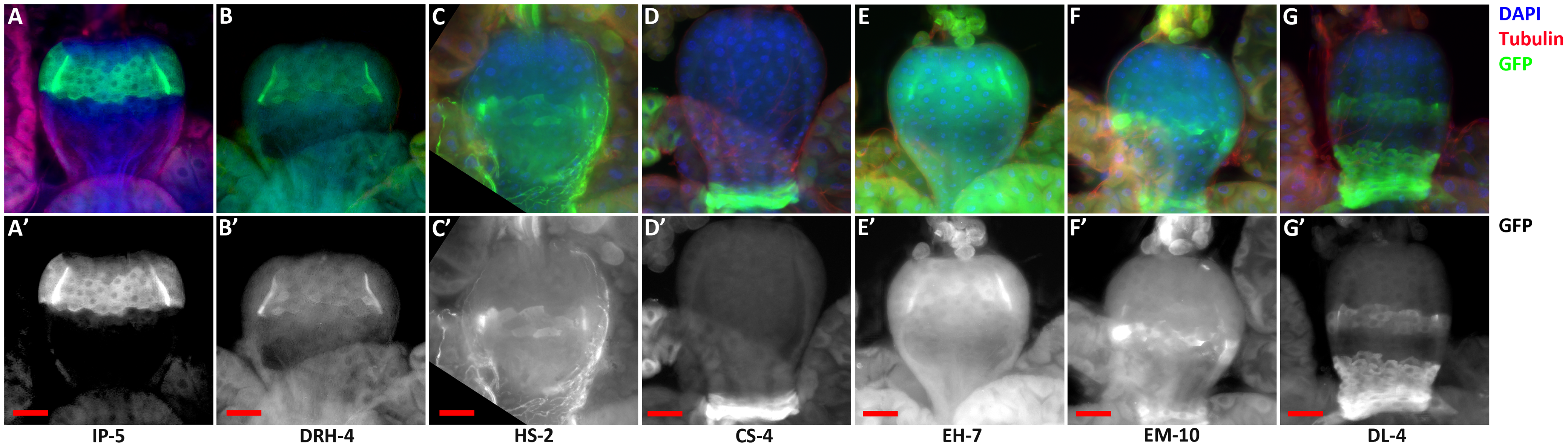

Supplement: Supplemental Material [file supp_g3.116.031229_FigureS4.tif]

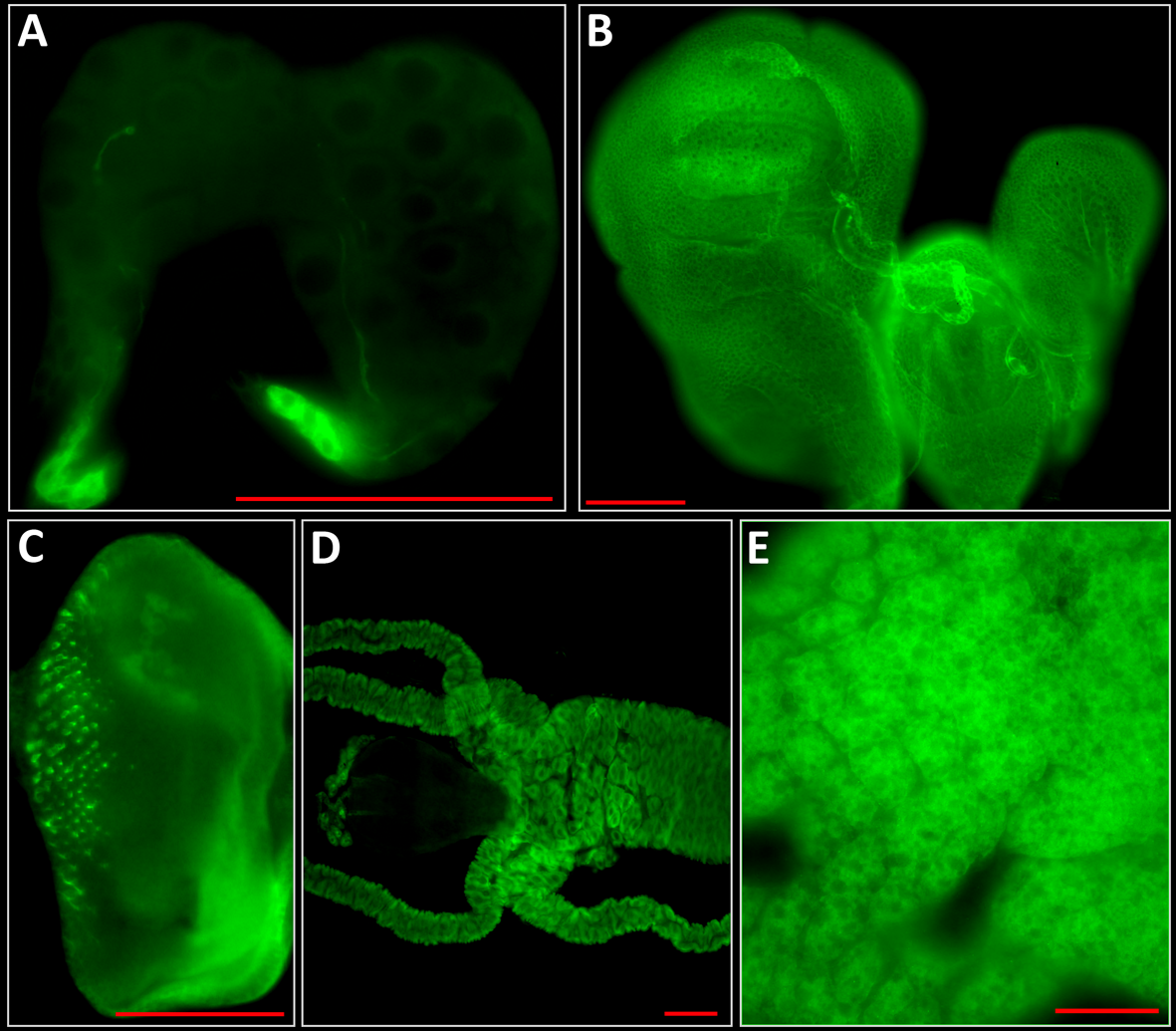

Supplement: Supplemental Material [file supp_g3.116.031229_FigureS5.tif]

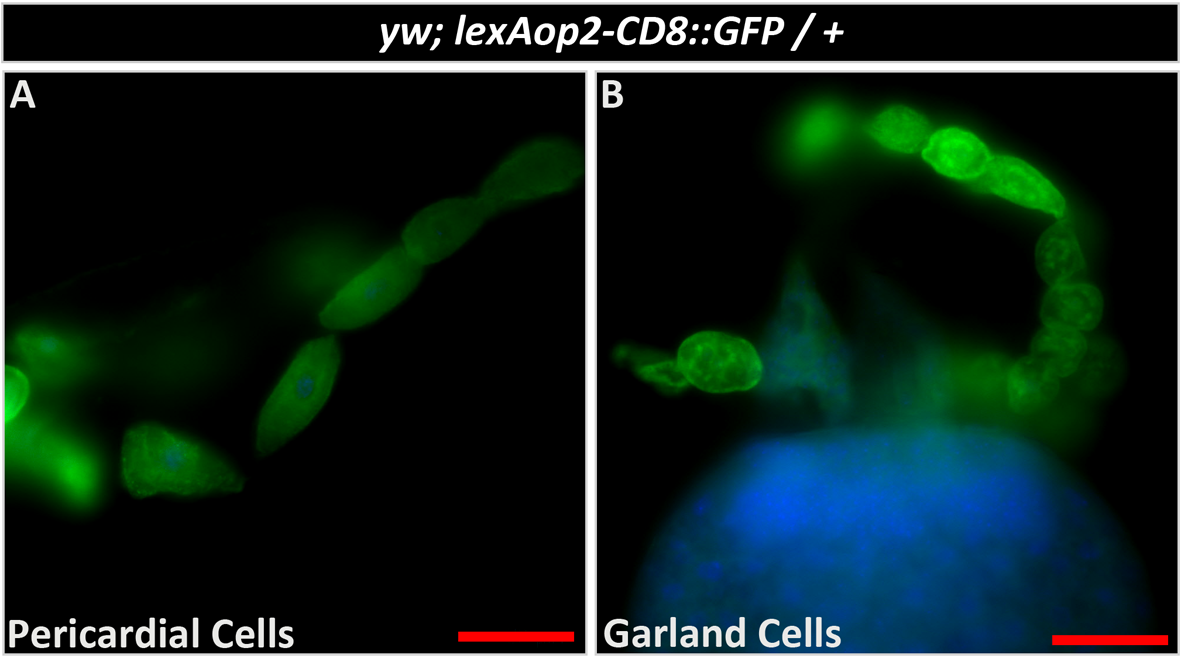

Supplement: Supplemental Material [file supp_g3.116.031229_FigureS6.tif]
